# Prevalence of central obesity and associated factors in Ethiopia: A systematic review and meta-analysis

**DOI:** 10.3389/fendo.2022.983180

**Published:** 2022-08-30

**Authors:** Kirubel Dagnaw Tegegne, Gebeyaw Biset Wagaw, Natnael Atnafu Gebeyehu, Lehulu Tilahun Yirdaw, Nathan Estifanos Shewangashaw, Nigusie Abebaw Mekonen, Mesfin Wudu Kassaw

**Affiliations:** ^1^ Department of Comprehensive Nursing, College of Medicine and Health Science, Wollo University, Dessie, Ethiopia; ^2^ Department of Pediatrics and Child Health Nursing, College of Medicine and Health Science, Wollo University, Dessie, Ethiopia; ^3^ Department of Midwifery, College of Medicine and Health Science, Wolaita Sodo University, Sodo, Ethiopia; ^4^ Department of Emergency Nursing, College of Medicine and Health Science, Wollo University, Dessie, Ethiopia; ^5^ Department of Midwifery, College of Medicine and Health Science, Wollo University, Dessie, Ethiopia; ^6^ School of Nursing, College of Health Science, Woldia University, Woldia, Ethiopia

**Keywords:** prevalence, central obesity, associated factors, meta-analysis, Ethiopia

## Abstract

**Introduction:**

Obesity is a global public health concern that is now on the rise, especially in low- and middle-income nations. Despite the fact that there are several studies reporting the prevalence of central obesity among adults in Ethiopia, there is a lack of a systematic review and meta-analysis synthesizing the existing observational studies. Therefore, this systematic review and meta-analysis aimed to determine the prevalence of central obesity and its associated factors in Ethiopia.

**Methods:**

Online libraries such as PubMed, Google Scholar, Scopus, Science Direct, and Addis Ababa University were searched. Data were extracted using Microsoft Excel and analyzed using STATA statistical software (v. 16). Forest plots, Begg’s rank test, and Egger’s regression test were all used to check for publication bias. To look for heterogeneity, I^2^ was computed, and an overall estimated analysis was carried out. Subgroup analysis was done by region and study setting. In addition, the pooled odds ratio for related covariates was calculated.

**Results:**

Out of 685 studies assessed, 20 met our criteria and were included in the study. A total of 12,603 people were included in the study. The prevalence of central obesity was estimated to be 37.31% [95% confidence interval (CI): 29.55–45.07]. According to subgroup analysis by study region and setting, the highest prevalence was observed in the Dire Dawa region (61.27%) and community-based studies (41.83%), respectively. Being a woman (AOR = 6.93; 95% CI: 3.02–10.85), having better socioeconomic class (AOR = 5.45; 95% CI: 0.56–10.34), being of age 55 and above (AOR = 5.23; 95% CI: 2.37–8.09), being physically inactive (AOR = 1.80; 95% CI: 1.37–2.24), being overweight (AOR = 4.00; 95% CI: 2.58–5.41), being obese (AOR = 6.82; 95% CI: 2.21–11.43), and having hypertension (AOR = 3.84; 95% CI: 1.29–6.40) were the factors associated with central obesity.

**Conclusion:**

The prevalence of central obesity was high in Ethiopia. Being a woman, having a higher socioeconomic class, being older, being physically inactive, being overweight or obese, and having hypertension were all associated. Therefore, it is vital for the government and health organizations to design and implement preventive measures like early detection, close monitoring, and positive reversal of central obesity in all patients and the general population. High-quality investigations on the prevalence of central obesity in the Ethiopian people are required to better understand the status of central obesity in Ethiopia.

**Systematic review registration:**

https://www.crd.york.ac.uk/PROSPERO, identifier: CRD42022329234.

## Background

Obesity is a serious and pressing public health issue. Over the last few decades, the prevalence has steadily increased, with rates nearly tripling since 1975 to the point where 30% of the world’s population are overweight or obese (1). In 2016, an estimated 1.9 billion adults worldwide were overweight, about 650 million of them being obese. Obesity is becoming a global public health concern, especially in low- and middle-income countries (LMICs) (2). Every year, 1.8 million individuals die prematurely as a result of noncommunicable diseases, in which obesity plays a key role (3).

Despite the fact that obesity was previously thought to be an issue only in high-income countries, recent reports reveal a dramatic increase in overweight and obesity in various LMICs (4). Whereas obesity in higher-income nations has been plateauing since the mid-2000s, it has been quickly increasing in LMICs, especially in numerous African countries, during the same time period (5). Obesity is common in Ethiopia, with 30% in Addis Ababa (6) and 28.5% in Hawasa, according to community-based surveys (7).

Obesity is a multifactorial medical condition characterized by complex pathogenesis involving biological (8), psychosocial (9), socioeconomic (10), and environmental factors (11, 12) and heterogeneity in the pathways and mechanisms by which it leads to adverse health outcomes (13, 14). The World Health Organization (WHO) defines central obesity (abdominal obesity) as a waist circumference (WC) of greater than 94 and 80 cm for men and women, respectively (15). Overall obesity and central obesity have a strong correlation. WC is an indicator of central obesity, which is linked with cardiometabolic diseases and cardiovascular diseases (CVDs) and is predictive of mortality (16, 17). Despite overall obesity being established as a cardiometabolic risk factor, central obesity is the strongest predictor of this risk regardless of the body mass index (BMI) (18, 19). In a recent study, it 7was discovered that WC had a better integrated discrimination index than the BMI in both men (6.9% versus 3.2%) and women (9.6% versus 9.2%) (20). Although High Density Lipoprotein cholesterol, High-Density Lipoprotein Cholesterol (HDL-C), hypertension, and diabetes increase the risk of CVD, central obesity remained the single significant factor for the risk after controlling for these factors. This implies that central obesity is the primary target for primary prevention of CVD. Furthermore, central obesity has been linked to a variety of health issues, including stroke (21), type 2 diabetes mellitus (22), hypertension (23), cancers, and all-cause mortality (24).

In Ethiopia, numerous cross-sectional studies have investigated the prevalence of central obesity and its associated factors among adults. These small and fragmented studies reported that central obesity among adults varied from 16.45% in Northern Ethiopia (25) to 76.1% in Eastern Ethiopia (26). These studies are small in sample size and confined in a certain geographic area, which cannot show the country-level prevalence of central obesity. Thus, a systematic review and meta-analysis is required as it provides a comprehensive overview of central obesity as a country-level burden. Given that there is no previous systematic review conducted, we aim to perform a systematic review and meta-analysis of prevalence of central obesity and its associated factors in Ethiopia. We pooled central obesity prevalence estimates from different regions of Ethiopia and analyzed the prevalence and associated factors of central obesity among Ethiopian adults. Estimating the country-level prevalence of central obesity and identifying the associated factors is crucial as it may inform strategy design and policy-making to mitigate the burden through health education, screening, and early intervention.

## Methods

This systematic review and meta-analysis study was conducted to determine the pooled prevalence of central obesity and its associated factors in Ethiopia using the standard PRISMA checklist guideline (27) (**Table S1**). This systematic review and meta-analysis is registered in PROSPERO under a registration number of CRD42022329234 and can be accessed at https://www.crd.york.ac.uk/PROSPERO.

### Search strategy

International online databases (Pub Med, Science Direct, Scopus, and Google Scholar) were used to search articles on the prevalence of central obesity. We also retrieved gray literature from Addis Ababa University’s online research institutional repository. The search string was established using “AND” and “OR” Boolean operators. The following core search terms and phrases with Boolean operators were used to search related articles: [(prevalence) OR (magnitude) OR (proportion) AND (central obesity) OR (abdominal obesity) OR (abdominal adiposity) OR (central fatness) OR (visceral obesity) OR (visceral adiposity) OR (metabolic syndrome) OR (metabolic risk factor) OR (cardiometabolic risk factor)) OR (waist circumference) AND (factors) OR (determinants) OR (predictors) AND (adults) OR (elders) OR (geriatrics) AND (Ethiopia)]. Search terms were based on PICO principles to retrieve relevant articles through the databases mentioned above. The search period was from 1 April 2022 to 5 May 2022.

### Outcome measurement

#### Central obesity

All adults with WC greater than 94 and 80 cm for men and women, respectively, were considered to have central obesity (15).

### Inclusion and exclusion criteria

This meta-analysis includes studies that reported the prevalence of central obesity in adults as study participants, only English language publications, both published and unpublished studies with full text available for search, and studies that took place in Ethiopia. Studies published between 1 January 2000 and 20 March 2022 were included. Studies that reported duplicated sources, qualitative studies from developed countries, studies with unreported outcome of interest, and articles without full text available were excluded from this systematic review and meta-analysis.

### Quality assessment

Two authors (KDT and NAG) independently appraised the standard of the studies using the Joanna Briggs Institute (JBI) standardized quality appraisal checklist (28). The disagreement raised during the quality assessment was resolved through a discussion led by the third author (GBW). Finally, the argument was solved and an agreement was reached. The critical analysis checklist has eight parameters with yes, no, unclear, and not applicable options. The parameters involve the following questions:

1. Were the criteria for inclusion in the sample clearly defined?
2. Were the study described the study setting and participants in detail?
3. Did the exposure measure the result validly and reliably?
4. Were the main objective and standard criteria used to measure the event?
5. Were confounding factors identified?
6. Did the study use strategies to deal with confounders?
7. Did the study use valid and reliable outcome measurement?
8. Was the statistical analysis appropriate?

Studies were considered of low risk when they scored 50% and above on the quality assessment indicators, as reported in a **supplementary file** (**Table S2**).

### Risk of bias assessment

Two authors (KDT and NAG) independently assessed included studies for risk of bias through the bias assessment tool developed by Hoy et al. (29), consisting of 10 items that assess four domains of bias and internal and external validity. Any disagreement raised during the risk of bias assessment was resolved through a discussion led by the third author (GBW). Finally, the argument was solved and an agreement reached. The first four items (items 1–4) evaluate the presence of selection bias, nonresponse bias, and external validity. The other six items (items 5–10) assess the presence of measuring bias, analysis-related bias, and internal validity. Therefore, studies that received “yes” for eight or more of the 10 questions were classified as “low risk of bias”. Studies that received “yes” for six to seven of the 10 questions were classified as ”moderate risk”, whereas studies that received “yes” for five or fewer of the 10 questions were classified as “high risk”, as reported in a **supplementary file** (**Table S3**).

### Data extraction

Microsoft Excel spreadsheet (2016) and STATA version 16 software were utilized for data extraction and analysis, respectively. Two authors (KDT and GBW) independently extracted all relevant data using a standardized JBI data extraction format. The disagreement raised during data extraction was resolved through a discussion led by the third author (LTY). Finally, the argument was solved and an agreement reached. The data automation tool was not used due to this study’s absence of the paper form (manual data). The name of the first author, the year of publication, the study region, the study setting, the study design, the prevalence of central obesity, the sample size, and the quality of each paper were extracted.

### Data analysis

After extracting all relevant findings in a Microsoft Excel spreadsheet, the data were exported to STATA software version 16 for analysis. The pooled prevalence of central obesity was computed using a 95% confidence interval (CI). Publication bias was checked by a funnel plot and more objectively through Begg’s and Egger’s regression tests (30), and a *p*-value less than 0.05 indicates that it is statistically significant (31). The presence of between-study heterogeneity was checked by using the Cochrane Q statistic. This heterogeneity between studies was quantified using I^2^, in which values of 25%, 50%, and 75% represented low, medium, and high heterogeneity, respectively (32). We used a forest plot to visually assess the presence of heterogeneity, which, as presented at a high-level random-effects model, was used for analysis to estimate the overall prevalence of central obesity. Subgroups were classified according to study setting (community setting versus institutional setting) and region (between 10 regions). Sensitivity analysis was executed to see the effect of a single study on the overall prevalence of the meta-analysis estimate. The findings of the study were presented in the form of text, tables, and figures.

## Results

### Study selection

**Figure 1:**
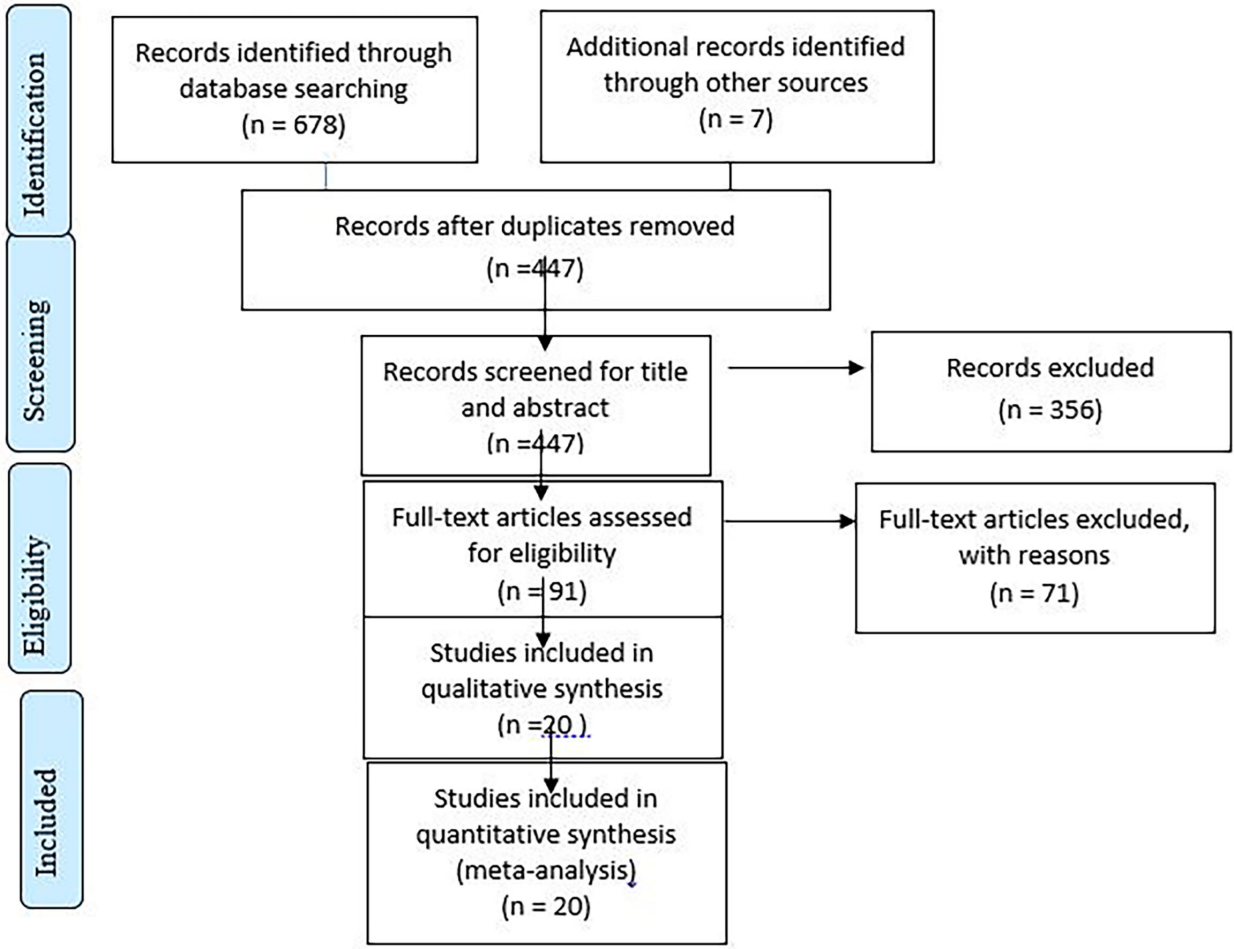
Flow chart illustrating the process of search and selection of studies included in the present systematic review and meta-analysis.

The literature search has yielded 685 records through electronic databases of PubMed, Scopus, Google Scholar, Science direct, and online research repository home. After duplicates were removed, 447 articles remained. Then, 356 studies were excluded after reviewing for full title and abstracts from the remaining 447 studies. Therefore, 91 full-text studies were assessed for eligibility criteria, which further excluded 71 studies due to unreported outcome of interest and being from different study populations or areas. Finally, 20 articles were included as the criteria for this systematic review and meta-analysis study (**Figure 1**).

### Characteristics of included studies

All included studies were employed by cross-sectional study design. Of these, five studies were community-based, whereas 15 were institutional-based cross-sectional studies: six studies in Amhara (25, 33–37), five studies in Addis Ababa (38–42), three studies in Tigray (43–45), two studies in Oromia (46, 47), two studies in Dire Dawa (26, 48), one study in Southern Nations Nationalists and Peoples Region (49), and one study in Harari (50). The sample sizes ranged from 225 to 1,935. The prevalence of central obesity ranges from 16.45 to 76.1. All studies were assessed by using the JBI quality appraisal checklist. Finally, 20 cross-sectional studies were evaluated, and all received a quality score of 75% or above on the quality scale, indicating that they are of low risk and included in the analysis (**Table 1**).

**Table 1:** Summary characteristics of studies included in the meta-analysis.

Author	Year	Region	Setting	Study design	Sample size	Prevalence	Quality
Samuel et al.	2021	Amhara	Community	Cross-sectional	802	16.45	Low risk
Zeleke et al.	2021	Addis Ababa	Institutional	Cross-sectional	450	22	Low risk
Adnan et al.	2021	Addis Ababa	Institution	Cross-sectional	353	71.67	Low risk
Balamurugan et al.	2020	Amhara	Institution	Cross-sectional	381	33.6	Low risk
Bayise et al.	2021	Oromia	Community	Cross-sectional	457	28.44	Low risk
Meseret et al.	2020	Amhara	Institution	Cross-sectional	773	37.64	Low risk
Ephrem et al.	2022	Dire Dawa	Community	Cross-sectional	611	76.1	Low risk
Fitsum et al.	Un-pub	Addis Ababa	Institutional	Cross-sectional	1,230	49.43	Low risk
Endris et al.	2016	Oromia	Institutional	Cross-sectional	225	16.88	Low risk
Gebreamlak et al.	2019	Tigrai	Institutional	Cross-sectional	419	30.54	Low risk
Lemlem et al.	2018	Tigrai	Institutional	Cross-sectional	1,380	42.68	Low risk
Melkam et al.	2020	Dire Dawa	Community	Cross-sectional	872	46.44	Low risk
Tran et al.	2011	Addis Ababa	Institutional	Cross-sectional	1,935	20.62	Low risk
Samrawit et al.	2019	Addis Ababa	Institutional	Cross-sectional	325	19.38	Low risk
Mequanenet et al.	2018	Amhara	Institutional	Cross-sectional	256	18.75	Low risk
Gebremedhin et al.	2021	Tigrai	Community	Cross-sectional	266	41.72	Low risk
Abouma et al.	2021	Harari	Institutional	Cross-sectional	1,164	46.82	Low risk
Belete et al.	2018	Amhara	Institutional	Cross-sectional	159	61	Low risk
Belaynesh et al.	2014	Amhara	Institutional	Cross-sectional	300	41.66	Low risk
Tesfaye et al.	2020	SNNP	Institutional	Cross-sectional	245	24.89	Low risk

## Meta-analysis

### Prevalence of central obesity in Ethiopia

**Figure 2:**
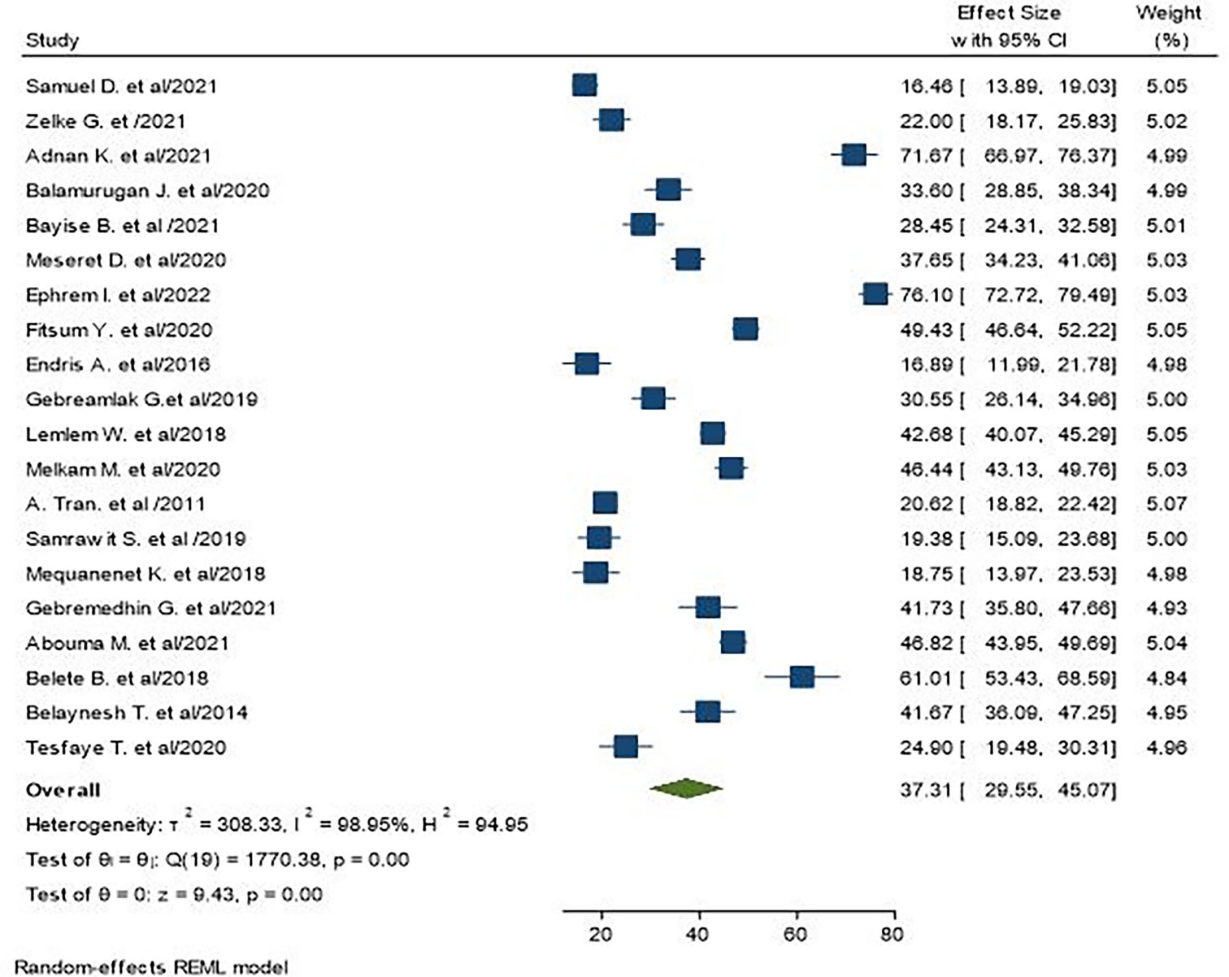
The pooled prevalence of central obesity among adult populations in Ethiopia.

The random-effects pooled prevalence of **central obesity** in Ethiopia was 37.31% (95% CI: 29.55–45.07), with significant heterogeneity observed between studies (I^2^ = 98.95%, P < 0.00) (**Figure 2**).

### Subgroup analysis

**Figure 3:**
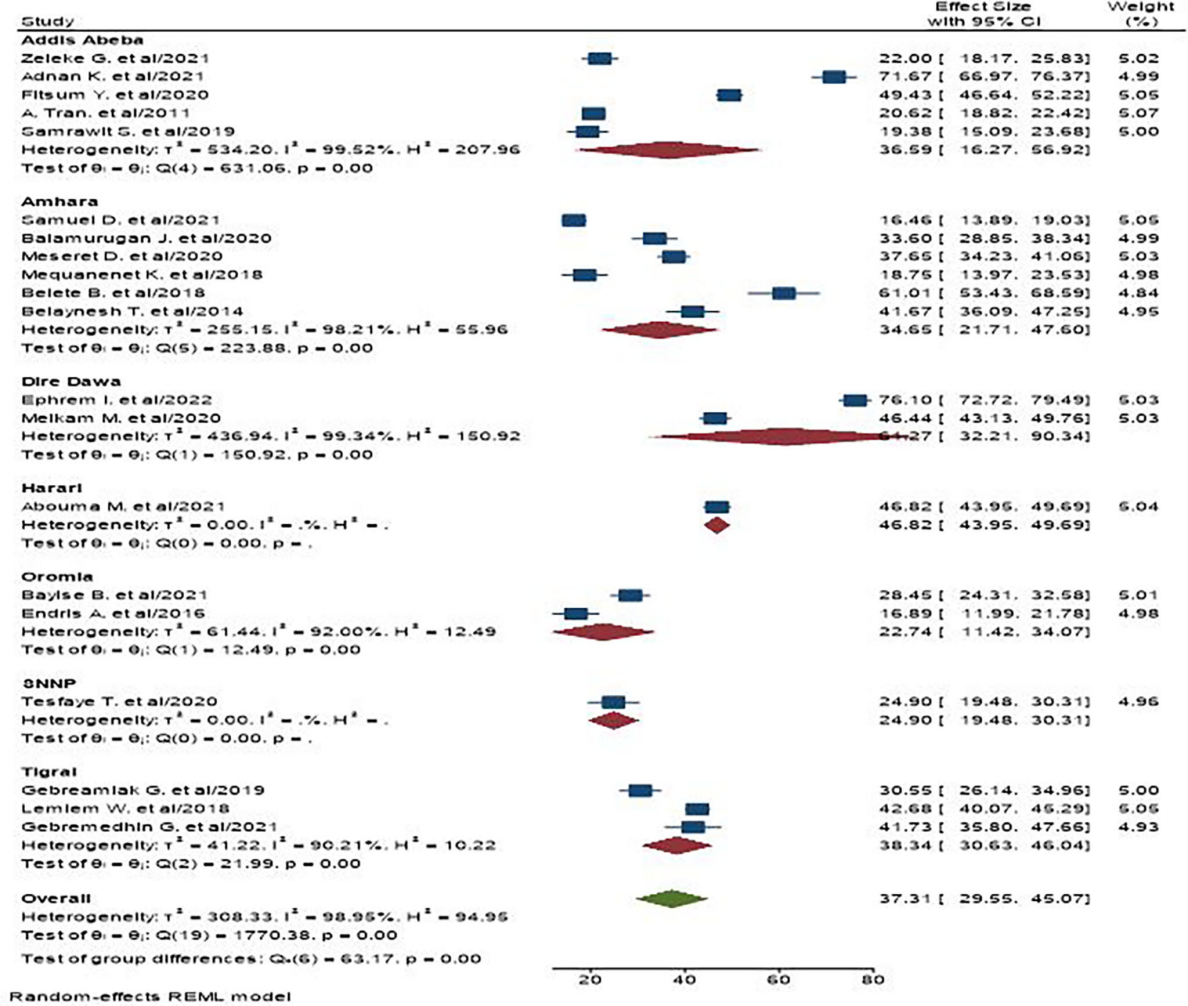
The pooled prevalence of central obesity among adult populations based on region the study is conducted in Ethiopia.

**Figure 4:**
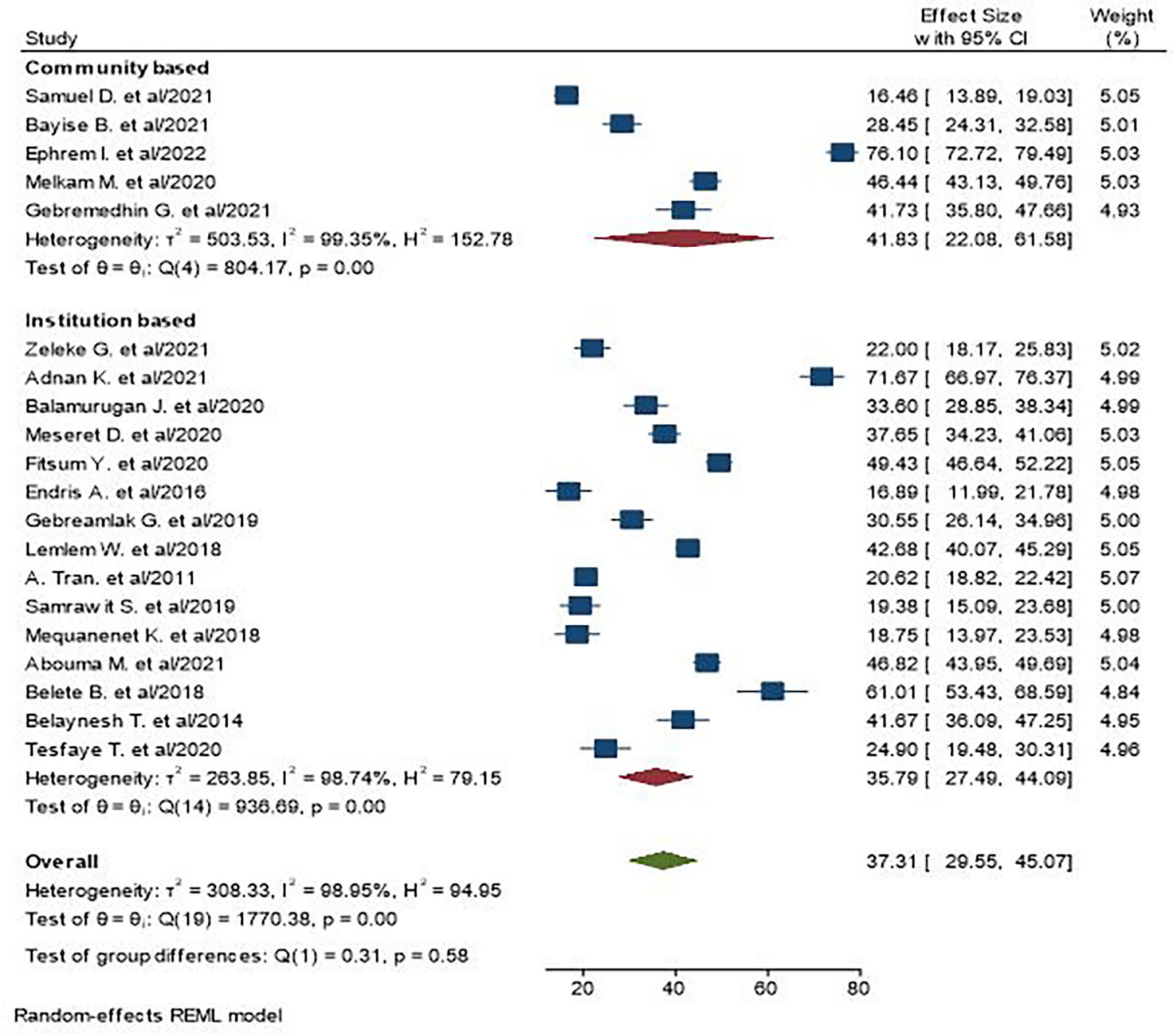
The pooled prevalence of central obesity among adult populations based on setting in Ethiopia.

We observed high heterogeneity between studies (I^2^ = 98.95%). As a result, subgroup analysis was conducted on the basis of study region and setting. As per the region, the highest pooled prevalence of central obesity was found at 61.27% in Dire Dawa, and in the context of study setting, the pooled prevalence in community-based studies appeared to be higher, i.e., 41.83% (**Figures 3**, **4**).

### Publication bias

**Figure 5:**
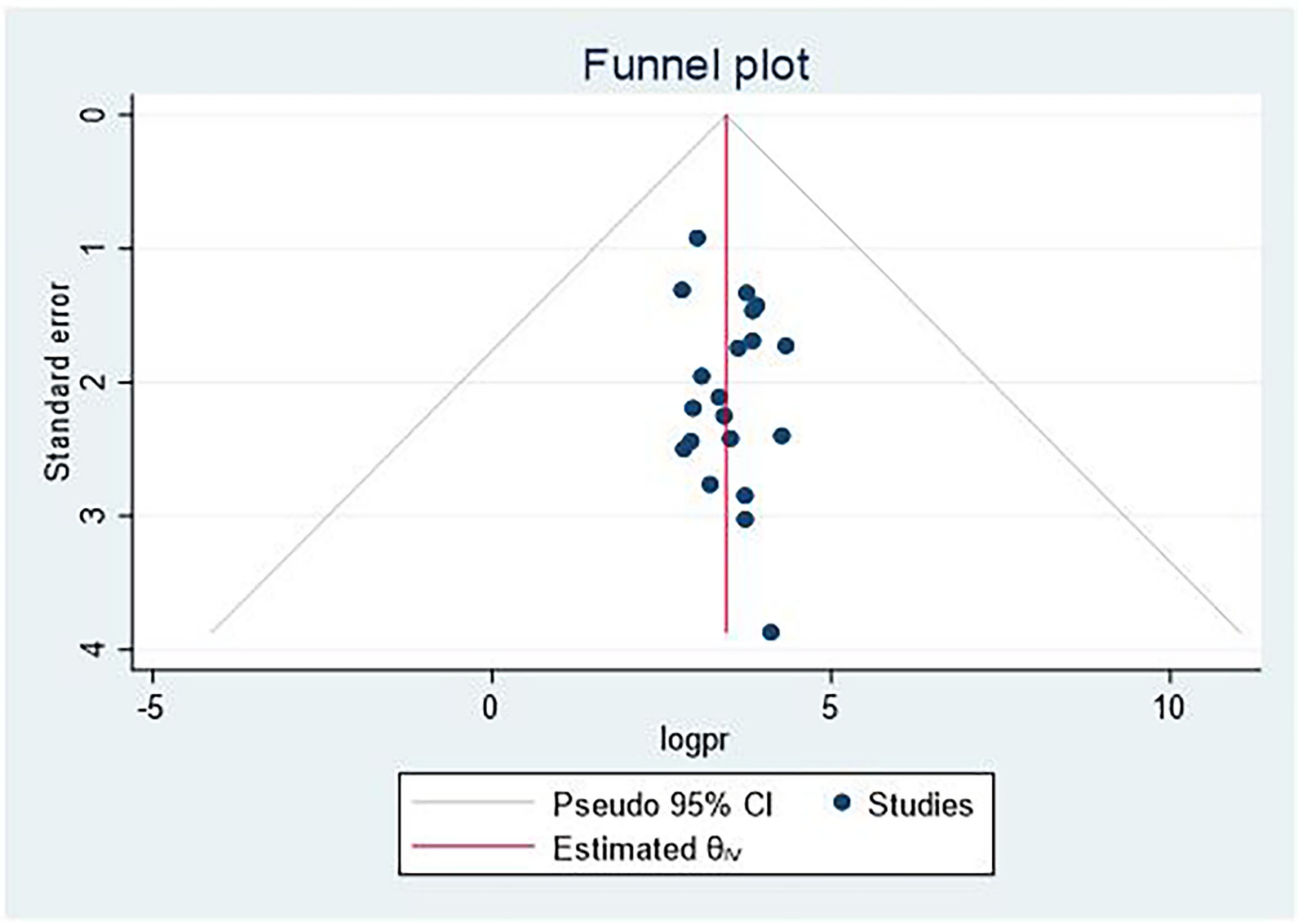
Funnel plot for the publication bias.

**Figure 6:**
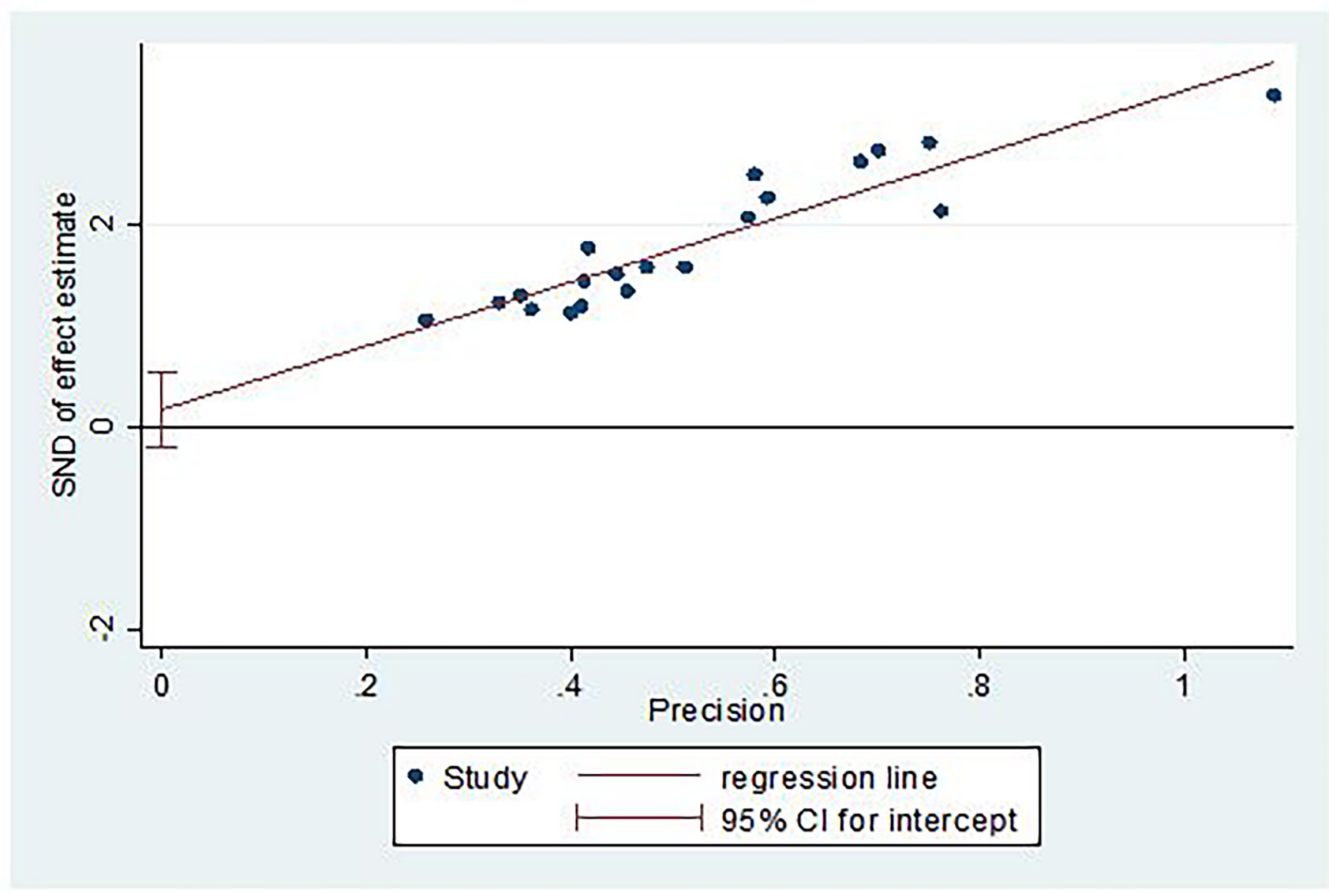
Egger test for small study effects.

The presence of publication bias was checked using funnel plot visualization and Egger’s and Begg’s regression tests (P < 0.05). Egger’s and Begg’s tests both revealed no statistical evidence of publication bias for prevalence of central obesity (P = 0.5432 and P = 0.9225), respectively (**Figures 5**, **6**).

### Sensitivity analysis for the studies included in central obesity

**Figure 7:**
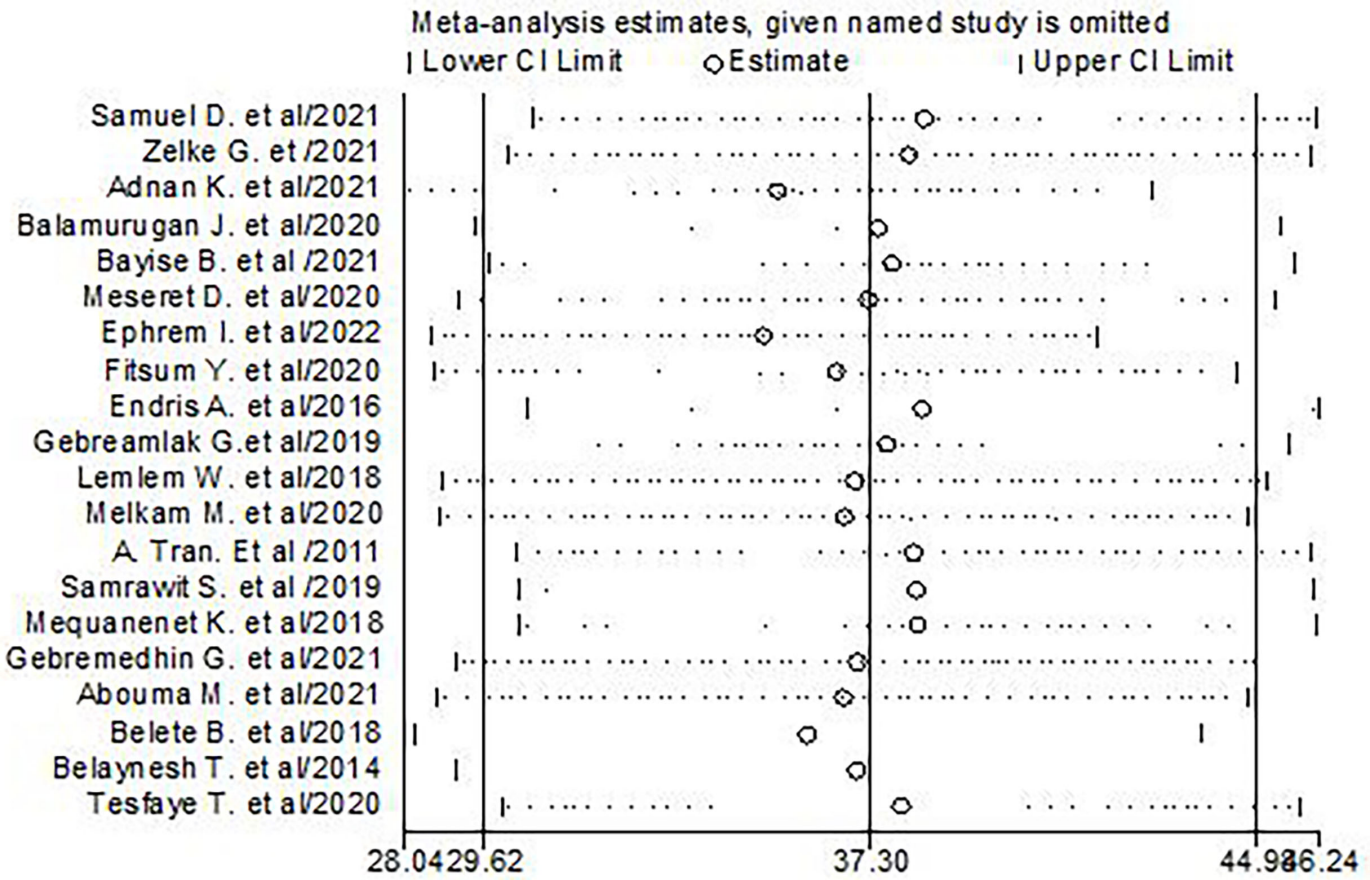
Results of sensitivity analysis of the 20 studies in the meta-analysis of central obesity.

To check the individual effect of included studies on the pooled prevalence of central obesity in Ethiopia, sensitivity analysis was performed using a random effect model, and the result revealed that no single study influenced the pooled prevalence of central obesity among adults. The pooled estimated prevalence of central obesity was estimated from 35.22 (28.59, 41.85) to 38.4 (30.64, 46.16) after omission of a single study (**Figure 7**).

### Factors associated with central obesity in Ethiopia

There are many factors associated with central obesity among adults in primary studies, but variables reported as a significant factor of central obesity in at least two primary studies were included in this meta-analysis. As a result, being a woman, having high socioeconomic status, being of age 55 and above, being physically inactive, being overweight or obese, and having hypertension are factors associated with central obesity.

As a result, being a woman (AOR = 6.93; 95% CI: 3.02–10.85; I^2^ = 99.65%, P = 0.00) is nearly seven times more likely to be obese compared with their counterparts. The odds of central obesity among people with higher socioeconomic status (AOR = 5.45; 95% CI: 0.56–10.34; I^2^ = 98.59%, P = 0.00) were five times more likely than those with lower socioeconomic status. The finding of our study showed that the odds of central obesity among older people of age greater than 55 (AOR = 5.23; 95% CI: 2.37–8.09; I^2^ = 96.86%, P = 0.00) were five times more than those who are of younger age. Moreover, this study showed that the prevalence of central obesity among people who are physically inactive (AOR = 1.80; 95% CI: 1.37–2.24; I^2^ = 41.76, P = 0.00) was 1.8 times more than adults who are physically active. The odds of central obesity of adults who are overweight (AOR = 4.00; 95% CI: 2.58–5.41; I^2^ = 0.00%, P = 0.00) were four times more likely than adults who are not overweight. Our finding also showed that the prevalence of central obesity among obese adults (AOR = 6.82; 95% CI: 2.21–11.43; I^2^ = 94.00%, P = 0.00) was 6.8 times more likely than adults who are not obese. Moreover, adults with hypertension (AOR = 3.84; 95% CI: 1.29–6.40; I^2^ = 95.75%, P = 0.00) were 3.8 times more likely than adults who are not overweight (**Table 2**).

**Table 2:** Summary estimate of OR for central obesity–associated factors.

Variables	Intervention	Comparator	Included Studies	OR (95% CI)	Pooled OR (95% CI)	I^2^
Gender	Female	Male	Samuel et al.	13.3 (12.66–13.94)	6.93 (3.02–10.85)	99.65
			Baysie et al.	5.59 (4.95–6.23)		
			Meseret et al.	9.62 (8.93–10.31)		
			Ephrem et al.	2.52 (2.08–2.96)		
			Melkam et al.	3.65 (3.31–3.99)		
Socioeconomic status	Higher	Lower	Samuel et al.	2.95 (2.06–3.84)	5.45 (0.56–10.34)	98.59
			Baysie et al.	7.94 (7.19–8.69)		
Age	Age>= 55	Age<55	Samuel et al.	3.8 (2.77–4.83)	5.23 (2.37–8.09)	96.86
			Baysie et al.	8.16 (7.23–9.09)		
			Ephrem et al.	3.75 (3.05–4.45)		
Physical activity	Physically inactive	Physically active	Ephrem et al.	2.05 (1.54–2.56)	1.8 (1.37–2.24)	41.76
			Fitsum et al.	1.6 (1.16–2.04)		
Body weight	Overweight	Normal	Samuel et al.	4.87 (1.81–7.93)	4.0 (2.58–5.41)	0.00
			Adnan et al.	3.5 (1.44–5.56)		
			Melkam et al.	4.15 (1.63–6.67)		
Body weight	Obese	Normal	Samuel et al.	9.29 (7.13–11.45)	6.82 (2.21–11.43)	94.00
			Melkam et al.	4.58 (3.92–5.24)		
Blood pressure	Hypertensive	Normal	Samuel et al.	2.53 (1.72–3.34)	3.84 (1.29–6.40)	95.75
			Zeleke et al.	5.14 (4.46–5.82)		

## Discussion

This review was conducted to determine the pooled prevalence of and factors for central obesity among adults aged 18 years and older in Ethiopia. To the best of our knowledge, this systematic review and meta-analysis is the first English-language study on the prevalence of central obesity among Ethiopian adults. In this meta-analysis, 20 articles with a total of 12,603 study subjects were included. The overall prevalence of central obesity in this study was 37.31% (95% CI: 29.55–45.07).

This higher figure of central obesity attributed to increased gender differences (36, 51), educational attainment and socioeconomic status (52), physical inactivity (26), and other diseases such as diabetes and hypertension (25, 53). Consistent with our result, the global prevalence of central obesity was reported to be 41.5% (54). Our results are likewise comparable to those reported in Nigeria (39%) (55) and China (37.6%) (56). One study among the population of 25–64 years old found that 22.5% of adults were centrally obese (57). Variations in the study population and geographical area can explain the disparity. Other studies such as those by Eyitayo et al. showed 67% (58)—a higher prevalence of central obesity compared with ours, which is 37.31% (95% CI: 29.55–45.07). A higher socioeconomic level and associated unhealthy lifestyle in South Africa relative to Ethiopia might explain the difference. Higher socioeconomic status is linked with the occurrence of central obesity (59).

A wide range of central obesity prevalence was found among the included studies, which could be attributable to the high heterogeneity of the samples included in this review. As a result, we further performed subgroup analyses by study region and setting related to central obesity. Thus, on the basis of region, Dire Dawa has the highest prevalence of central obesity, which is 61.27%. Dire Dawa is one of the metropolitan cities in the country, where industrialization and urbanization are higher compared with the rural areas in the country. Individuals who live in urban areas and industrialized towns are at a higher risk of getting central obesity due to their overconsumption of processed and energy-dense foods more frequently than that of the rural people (60, 61). Moreover, the prevalence of central obesity in a community setting is higher than institutions. This can be explained, in part, by people with obesity in the community who are less likely to seek obesity care in health institutions unless they have obesity-related diseases (62). Sample size and study population variations can be additional contributors for the difference between the two settings.

We found that being a woman is a significant predictor of central obesity; women appear to be nearly seven times more likely to develop central obesity. This result is in line with various previous studies (54, 55, 63, 64). Biological differences where women have higher body fat composition compared to men could be the reason for the variation between females and males. Cultural and social restrictions imposed on women might also explain this gender difference. Women tend to be less physically active due to lower educational level, sedentary lifestyle, and higher household activities engagement (65, 66). Furthermore, sex hormones and the effect of menopause could explain the difference between men and women. Central obesity seems to be linked with low levels of testosterone as the hormone increases fat consumption and thus decreases central obesity (67–69).

Previous studies have shown that aging is strongly associated with the prevalence of central obesity (54, 57, 64). The present study results also show the natural pattern of central obesity increase with age, and the highest prevalence of central obesity was seen in people over 55 years. Aged people become often less active, which contributes to reduced energy expenditure, and this leads to fat accumulation in the abdominal area (70). In addition, our study revealed that people from higher socioeconomic status are more likely to be centrally obese, and this result is supported by studies conducted globally (54). Studies suggest that, in lower-income countries, people from higher socioeconomic status are more likely to be obese (71). Similarly, this may also be applied to central obesity. The high prevalence of central obesity from higher socioeconomic family underscores the importance for policy-makers and clinicians to design strategies favorable for patients and the general population. Physical inactivity is the other critical factor that drives people to be centrally obese. Physical inactivity has become increasingly popular over the past few decades and may have contributed to the upsurge of central obesity. A study from the global perspective (54) also mentioned that physical inactivity accounts for the problem of central obesity. The current low levels of physical activity attributed to rapid urbanization and associated sedentary behaviors in working and domestic environments (72).

This systematic review showed that being overweight or obese is associated with central obesity. We found that people who are overweight and obese are 4 and 6.8 times more likely to be centrally obese, respectively. People who are overweight or obese can gain extra fat throughout the whole body, and this includes fat accumulation in the abdominal area, named as central obesity. It is also evident that being hypertensive is associated with central obesity. As shown in our study, patients who are hypertensive are 3.8 times more likely to be centrally obese compared with those without hypertension. Activation of the renin-angiotensin system in hypertension is well known and plays a role in insulin resistance, which contributes to central obesity (73). This implies that designing health education strategies and early control of hypertension by the concerned bodies is a priority. Overall, the implications for policy-makers, health professionals, and supporting agencies are to encourage the prevention of central obesity, weight loss, and more physical activity in women and older population, and to manage other comorbidities such as hypertension.

## Conclusion

In conclusion, our study demonstrated that the prevalence of central obesity is increasing. Moreover, the pooled prevalence of central obesity varies on the basis of study settings and regions. Being a woman, having high socioeconomic status, being of age 55 and above, being physically inactive, being overweight or obese, and having hypertension were factors associated with central obesity. Therefore, it is vital for the government and health organizations to design and implement preventive measures like early detection, close monitoring, and positive reversal of central obesity in all patients and the general population. High-quality investigations on the prevalence of central obesity in the Ethiopian people are required to better understand the status of central obesity in Ethiopia.

### Strength and limitations

This study has some limitations. First, articles were restricted to only being published in the English language, which may result in the exclusion of other articles. Second, the meta-analyses revealed high heterogeneity in the estimated pooled prevalence. Sample size variations, geographical areas, and other different factors in the studies might explain the high heterogeneity of estimates observed in the current study. Therefore, the results of this meta-analysis should be interpreted cautiously. Third, all included studies were cross-sectional, which might affect the outcome variable because of other confounding factors.

This study has strengths. First, compressive electronic online international search engines were used. Second, both published and unpublished (gray literature) studies were included in this review. Third, factors associated with central obesity were identified.

## Data availability statement

The original contributions presented in the study are included in the article/**Supplementary Material**. Further inquiries can be directed to the corresponding author.

## Author contributions

KT conceptualized the study; KT, GB, and LT contributed during data extraction and analysis; KT, GB, and NS interpreted the results; KT and GB prepared the first draft; KT, NG, GB, and LT contributed during the conceptualization and interpretation of results and substantial revision; KT, NM, NS, GB, MK, and LT revised and finalized the final draft manuscript. All authors read and approved the final version of the manuscript.

## Conflict of interest

The authors declare that the research was conducted in the absence of any commercial or financial relationships that could be construed as a potential conflict of interest.

## Publisher’s note

All claims expressed in this article are solely those of the authors and do not necessarily represent those of their affiliated organizations, or those of the publisher, the editors and the reviewers. Any product that may be evaluated in this article, or claim that may be made by its manufacturer, is not guaranteed or endorsed by the publisher.

## Abbreviations

BMI, body mass index; CVD, cardiovascular diseases; JBI, Joanna Briggs Institute; LMICs, low- and middle-income countries; PRISMA, Preferred Reporting Items for Systematic Reviews and Meta-Analyses; WC, waist circumference; WHO, World Health Organization.
