# Supplementary material for: Prevalence of central obesity and associated factors in Ethiopia: A systematic review and meta-analysis
Source: Front Endocrinol (Lausanne). 2022 Aug 30;13:983180. doi: 10.3389/fendo.2022.983180 (PMC9468774; doi:10.3389/fendo.2022.983180)
Supplement: Supplementary file 2 [file Table_2.docx]

S 2 Table. Quality assessment for the included Studies

| Item | Clearly defined inclusion | Describe study setting and participant | Valid and reliable exposure measurement | Objective and standard criteria for measurement | Identified confounder | Strategies to deal with confounders | Valid and reliable outcome measurement | Appropriate statically analysis | No of ‘yes’ ‘ |
| --- | --- | --- | --- | --- | --- | --- | --- | --- | --- |
| Samuel D. et al | Yes | Yes | No | Yes | Yes | No | Yes | Yes | 6/8=75 |
| Zelke G. et | Yes | Yes | Yes | Yes | No | No | Yes | Yes | 6/8=75 |
| Adnan K. et al | Yes | Yes | No | Yes | Yes | No | Yes | Yes | 6/8=75 |
| Balamurugan J. et al | Yes | Yes | No | Yes | Yes | Yes | Yes | Yes | 7/8=87.5 |
| Bayise B. et al | Yes | Yes | No | Yes | Yes | Yes | Yes | Yes | 7/8=87.5 |
| Meseret D. et al | Yes | Yes | Yes | Yes | Yes | No | Yes | Yes | 7/8=87.5 |
| Ephrem I. et al | Yes | Yes | Yes | Yes | No | No | Yes | Yes | 6/8=75 |
| Fitsum Y. et al | Yes | Yes | No | Yes | Yes | Yes | Yes | No | 6/8=75 |

| Endris A. et al | Yes | Yes | Yes | Yes | No | Yes | Yes | No | 6/8=75 |
| --- | --- | --- | --- | --- | --- | --- | --- | --- | --- |
| Gebreamlak G.et al | Yes | yes | No | Yes | Yes | Yes | Yes | Yes | 7/8=87.5 |
| Lemlem W. et al | Yes | Yes | Yes | No | Yes | No | Yes | Yes | 6/8=75 |
| Melkam M. et al | Yes | Yes | Yes | Yes | Yes | Yes | Yes | No | 7/8=87.5 |
| A. Tran. Et al | Yes | Yes | Yes | Yes | Yes | Yes | No | Yes | 7/8=87.5 |
| Samrawit S. et al | Yes | Yes | No | Yes | No | Yes | Yes | Yes | 6/8=75 |
| Mequanenet K. et al | Yes | Yes | Yes | Yes | Yes | Yes | Yes | No | 7 /8=87.5 |
| Gebremedhin G. et al | Yes | Yes | No | Yes | Yes | No | Yes | Yes | 6/8=75 |
| Abouma M. et al | Yes | Yes | Yes | Yes | Yes | Yes | Yes | No | 7 /8=87.5 |
| Belete B. et al | Yes | Yes | Yes | Yes | Yes | Yes | Yes | No | 7 /8=87.5 |
| Belaynesh T. et al | Yes | Yes | No | Yes | Yes | No | Yes | Yes | 6/8=75 |
| Tesfaye T. et al | Yes | Yes | Yes | Yes | Yes | Yes | Yes | No | 7 /8=87.5 |
