# Supplementary material for: Prevalence of central obesity and associated factors in Ethiopia: A systematic review and meta-analysis
Source: Front Endocrinol (Lausanne). 2022 Aug 30;13:983180. doi: 10.3389/fendo.2022.983180 (PMC9468774; doi:10.3389/fendo.2022.983180)
Supplement: Supplementary file 3 [file Table_3.docx]

S 3 Table. Risk of bias of assessment for the cross-sectional studies

| Item | External validity | | | | Internal validity | | | | | |  | |
| --- | --- | --- | --- | --- | --- | --- | --- | --- | --- | --- | --- | --- |
|  | Representativeness s of the target population | Representativeness s of the sampling frame | Radom samplin g or census | Minimal responses e bias | Data were collect d directly | Acceptable e case definition used in the study | Valid and reliable measurement t | The same mode of data collection n for all study subject | Appropriate e length of prevalence period for parameter of interest | Appropriate numerators and denominator s of interest | No of yes | **Summ ary of risk of bias** |
| Samuel D. et al | Yes | Yes | No | Yes | Yes | No | Yes | Yes | Yes | Yes | 8 | Low-  risk |
| Zelke G. et | Yes | Yes | Yes | Yes | No | No | Yes | Yes | Yes | Yes | 8 | Loiw risk |
| Adnan K. et al | Yes | Yes | No | Yes | Yes | No | Yes | Yes | Yes | Yes | 8 | Low- risk |
| Balamurugan J. et al | Yes | Yes | No | Yes | Yes | Yes | Yes | Yes | Yes | Yes | 9 | Low – risk |
| Bayise B. et al | Yes | Yes | No | Yes | Yes | Yes | Yes | Yes | Yes | Yes | 9 | Low- risk |
| Meseret D. et al | Yes | Yes | Yes | Yes | Yes | No | Yes | Yes | Yes | Yes | 9 | Low- risk |
| Ephrem I. et al | Yes | Yes | No | Yes | Yes | Yes | Yes | Yes | Yes | Yes | 9 | Low- risk |
| Fitsum Y. et al | Yes | Yes | Yes | Yes | No | No | Yes | Yes | Yes | Yes | 8 | Low- risk |
| Endris A. et al | Yes | Yes | No | Yes | Yes | Yes | Yes | No | Yes | Yes | 8 | Low- risk |

| Gebreamlak G.et al | Yes | Yes | Yes | Yes | No | Yes | Yes | No | Yes | Yes | 8 | Low- risk |
| --- | --- | --- | --- | --- | --- | --- | --- | --- | --- | --- | --- | --- |
| Lemlem W. et al | Yes | Yes | Yes | Yes | No | No | Yes | Yes | Yes | Yes | 8 | Low-  risk |
| Melkam M. et al | Yes | yes | No | Yes | Yes | Yes | Yes | Yes | Yes | Yes | 9 | Low- risk |
| A. Tran. Et al | Yes | Yes | Yes | No | Yes | No | Yes | Yes | Yes | Yes | 8 | Low-  risk |
| Samrawit S. et al | Yes | Yes | Yes | Yes | Yes | Yes | Yes | No | Yes | Yes | 9 | Low-  risk |
| Mequanenet K. et al | Yes | Yes | Yes | Yes | Yes | Yes | No | Yes | Yes | Yes | 8 | Low- risk |
| Gebremedhin G. et al | Yes | Yes | No | Yes | Yes | Yes | Yes | Yes | Yes | Yes | 9 | Low risk |
| Abouma M. et al | Yes | Yes | Yes | Yes | Yes | Yes | Yes | Yes | No | No | 8 | Low- risk |
| Belete B. et al | Yes | Yes | Yes | Yes | Yes | Yes | Yes | No | No | Yes | 8 | Low- risk |
| Belaynesh T. et al | Yes | Yes | No | Yes | Yes | Yes | Yes | Yes | Yes | Yes | 9 | Low risk |
| Tesfaye T. et al | Yes | Yes | Yes | No | Yes | No | Yes | Yes | Yes | Yes | 8 | Low-  risk |
